# The sentiment of a virtual rock concert

**DOI:** 10.1007/s10055-022-00685-9

**Published:** 2022-08-23

**Authors:** Mel Slater, Carlos Cabriera, Gizem Senel, Domna Banakou, Alejandro Beacco, Ramon Oliva, Jaime Gallego

**Affiliations:** 1 Event Lab, Faculty of Psychology, University of Barcelona, Barcelona, Spain; 2 Institute of Neurosciences of the University of Barcelona, Barcelona, Spain

**Keywords:** Virtual reality, Sentiment analysis, Evaluation, Concert, Performance, Plausibility, Presence

## Abstract

We created a virtual reality version of a 1983 performance by Dire Straits, this being a highly complex scenario consisting of both the virtual band performance and the appearance and behaviour of the virtual audience surrounding the participants. Our goal was to understand the responses of participants, and to learn how this type of scenario might be improved for later reconstructions of other concerts. To understand the responses of participants we carried out two studies which used sentiment analysis of texts written by the participants. Study 1 (*n* = 25) (Beacco et al. in IEEE Virtual Reality: 538–545, 2021) had the unexpected finding that negative sentiment was caused by the virtual audience, where e.g. some participants were fearful of being harassed by audience members. In Study 2 (*n* = 26) notwithstanding some changes, the audience again led to negative sentiment—e.g. a feeling of being stared at. For Study 2 we compared sentiment with questionnaire scores, finding that the illusion of being at the concert was associated with positive sentiment for males but negative for females. Overall, we found sentiment was dominated by responses to the audience rather than the band. Participants had been placed in an unusual situation, being alone at a concert, surrounded by strangers, who seemed to pose a social threat for some  of them. We relate our findings to the concept of Plausibility, the illusion that events and situations in the VR are really happening. The results indicate high Plausibility, since the negative sentiment, for example in response to being started at, only makes sense if the events are experienced as actually happening. We conclude with the need for co-design of VR scenarios, and the use of sentiment analysis in this process, rather than sole reliance on concepts proposed by researchers, typically expressed through questionnaires, which may not reflect the experiences of participants.

**Supplementary Information:**

The online version contains supplementary material available at 10.1007/s10055-022-00685-9.

## Introduction

The traditional paradigm for the evaluation of a new method, application or scenario in virtual reality (VR) is to carry out an experimental study, with response variables as answers on Likert scale questionnaires and possibly some behavioural or physiological measures. The point is to understand how the responses vary between different levels of the factors of the experiment. For example, one of the earliest ever VR studies was concerned with transfer of training in a physical manipulation task from VR to real world performance (Kozak et al. 1993) and found that VR training offered no advantage compared to a group that received no training (in the particular system used at that time). More generally, a long-standing theme in the evaluation of VR experiences has been the concept of presence (the feeling of “being there”) in the place depicted by the VR (Sheridan 1992). Since this is a unique affordance of VR the achievement of high presence has been thought to be a fundamental goal of VR experiences. A stream of studies started in the early 1990s that analysed different factors that may contribute to presence, for example, Slater et al. (1995) examined how walking-in-place compared to point-and-click methods of moving through an environment influenced presence, and Barfield and Hendrix (1995) examined the impact of display update rate.

Presence is usually evaluated by questionnaire (Lessiter et al. 2001; Usoh et al. 2000; Witmer and Singer 1998), physiological responses (Meehan et al. 2002), breaks in presence (Slater and Steed 2000) or psychophysical approaches where factors can be varied in real time in order to find their optimal balance (Llobera et al. 2021; Slater et al. 2010). Research on presence is reviewed in (Sanchez-Vives and Slater 2005; Skarbez et al. 2018) with a meta-analysis concerned with factors found to influence presence in (Cummings and Bailenson 2016). However, the concept of presence has evolved and has been deconstructed into two orthogonal components (Slater 2009; Slater et al. 2022). Place Illusion (PI) refers to the illusion that participants have of being in the place depicted by the VR displays, even though they know that this is not true. The root of this is that perception should be based on the extent to which natural sensorimotor contingencies (O'Regan and Noë 2001a, 2001b) are afforded by the VR system. This refers to using the whole body for perception (e.g. head turns, looking around and underneath objects, turning the whole body, eye movements) resulting in the same changes in sensory input as in reality. For example, a stereo wide field-of-view head mounted display with head 6 degrees of freedom head tracking meets many of the requirements for natural sensorimotor contingencies for vision, and in the case of spatialized sound, for audition too. The second component of presence is referred to as Plausibility (Psi). This is the illusion that the events that are perceived to be happening in the VR are really happening, even though this is known not to be true. Psi depends on (i) Events in the VR responding to the actions of the participant (for example, a virtual character looks back when looked at), (ii) Events that spontaneously refer to the participant (e.g. a virtual character contingently looks at the participant and smiles), (iii) That where the VR depicts events or a situation that participants are quite familiar with in reality, that their expectations are met. Requirement (iii) Is often difficult to satisfy since it requires detailed domain knowledge by the application designers, and is complex in itself. For example, participants might well accept a VR with strange creatures or where normal physical laws are not obeyed—for example, in the case of 3D chess in VR where chess pieces fly through the air of their own accord (Slater et al. 1996)—but not accept a situation where some detail fails to meet expectations—for example, in our work on violence between soccer fans in a bar, our first rendition of the bar was rejected by participants on the grounds that a bar decorated in that way would never be visited by soccer fans (Rovira et al. 2009). Medical doctors experienced less Psi in an interview with virtual patients because they were unable to look up patient details on a virtual computer display that was on their virtual desk (Pan et al. 2016). Plausibility is probably the more difficult (and interesting) illusion to generate and has been increasingly studied, for example recently (Hofer et al. 2020) examined the relationship between PI and Psi with results suggesting their independence, (Galvan Debarba et al. 2020) studied the impact of different levels of body tracking on Psi using the psychophysics methodology of (Slater et al. 2010), the impact of virtual human character behaviour and other factors on Psi were considered in (Bergström et al. 2017; Skarbez et al. 2017).

The standard experimental paradigm and methods of measurement are appropriate when there are specific hypotheses in mind, or when we know what relationships we are interested in investigating. For example, whether spatialized sound is likely to result in greater scores on a presence questionnaire (Poeschl et al. 2013), or to examine how display latency influences presence (Meehan et al. 2003). However, in the case of a novel application where there is no or little prior knowledge about how participants may respond, or what factors may be important, this paradigm may be uninformative or even misleading. A questionnaire score can mask critical information.

We embarked on a new research field concerned with the recreation of historical rock concerts in VR. This was to be the most complex of scenarios that we had tackled up to date, and the reconstruction of past events such as this in VR although an immense challenge could also be useful for many applications, beyond rock concerts. The rock concert involved two different elements—the depiction of the band itself and the audience. It involved three major challenges. First, on the technical side the idea was to employ computer vision techniques (Beacco et al. 2020; Beacco et al. 2022; Gallego and Slater 2020) to extract the appearance and movements of the band players in 3D. The second challenge was to use agent-based models and crowd rendering to reconstruct a virtual audience, and to place the players and audience in a model of the theatre in which the concert took place. The sound from the original video at the basis of the reconstruction was used for the audio. Third, our scientific interest has been to explore how people would respond to the virtual concert—would they reject it because of the inevitable lack of realism? Would they join in dancing along with the audience? How much would they feel as if they were at an actual concert? and so on. Which factors might contribute to or detract from these? The particular performance on which we have focussed is from the 1983 Alchemy concert by Dire Straits playing “Sultans of Swing” at the Hammersmith Odeon in London (this was a personal choice and had no other significance). In our pilot study (Beacco et al. 2021) 25 participants were recruited online and from an overseas University class. The scenario involved a recreation of the Hammersmith Odeon, the players on stage modelled partially from a video of the live performance, and a virtual audience that surrounded the participant. The audience had a number of realistic male avatars standing in the immediate vicinity of the participant, which were created from photographs of men using our computer vision techniques so that they looked like actual people, and further away from the participant the audience members were standard graphics-based avatars, and further away still were impostors. The audience moved with the music though the dancing animations that were taken from online repositories of animations.

Since our questions were very open, exploring this quite new area of application, instead of questionnaires and behavioural measures, we used sentiment analysis. Sentiment analysis (Bakshi et al. 2016; Liu 2012) relies on prior classifications of millions of words in dictionaries which have been assigned positive or negative valence, we discuss this further below. Pieces of text then obtain a score, for example, as the average score over all the relevant words in the text. The major response variable was derived from a sentiment analysis of short essays that participants were asked to write immediately after their experience. This analysis led to the quite unexpected result that the virtual audience was more impactful than the actual performance by the band. In particular, some participants felt vulnerable, and alone amongst the audience, had a feeling of being stared at by audience members (even though this was not programmed to occur), and especially women felt that they would be on the receiving end of unwelcome approaches from the surrounding men. Such feelings were classified as “disturbing”. However, this also signified a high degree of Plausibility of the experience, since a prerequisite of feeling disturbed is that the events in question must be experienced as really happening, an automatic response, not a belief. High disturbance was associated with low sentiment scores. A second contributor to lower sentiment scores was a failure of expectations—examples being the band not interacting with the audience, or the drummer not visually beating in time to the sound of the drums. On the positive side, higher sentiment scores were associated with a feeling of immersion in the concert, people joining in with the dancing of those around them, spatial audio from the band, and the movement of the crowd around.

We refer to this as Study 1. An important overall conclusion from the results of Study 1 was that a standard VR experimental design, with a questionnaire asked after the experience with Likert scale questions, would never have picked up on these deeper findings about the responses of participants. For example, Plausibility may have scored highly, but the underlying disturbance associated with this, might never have been discovered.

Here we report the results of a second experiment (Study 2) of exposure to the VR concert with a number of changes:

1. To further examine the impact of the audience the virtual audience members were all depicted as female. This deliberately went to the other extreme compared to the first study. Would this lessen the chance of disturbance, especially as earlier reported amongst women participants?
2. All audience members visible to the participant were generated with the software Character Creator 3, being more pleasant and realistic than the earlier ones.
3. The movements of the audience members, depicted as dancing along with the music, were based on motion capture of a few individuals actually dancing in rhythm to the same music.
4. There were various small improvements to the portrayal and movement of the band members.

The overall goal of this paper is to introduce a new method for the analysis of how people responded to the concert scenario based on sentiment analysis, in order to discover those aspects of the scenario that might be improved for later versions, and to contribute our findings to the concept of presence (Place Illusion and more importantly Plausibility). Hence this paper is concerned almost wholly with evaluation and not with the technical aspects of how the scenario was created. Methods for the two studies are described in the next section, including a description of the sentiment analysis used. We then present results for Study 2, and then combine the data from both Studies and analyse those together. Conclusions about sentiment analysis, the concert scenario and the way forward are presented in Sect. 5.

## Methods

### The scenario

**Fig. 1:**
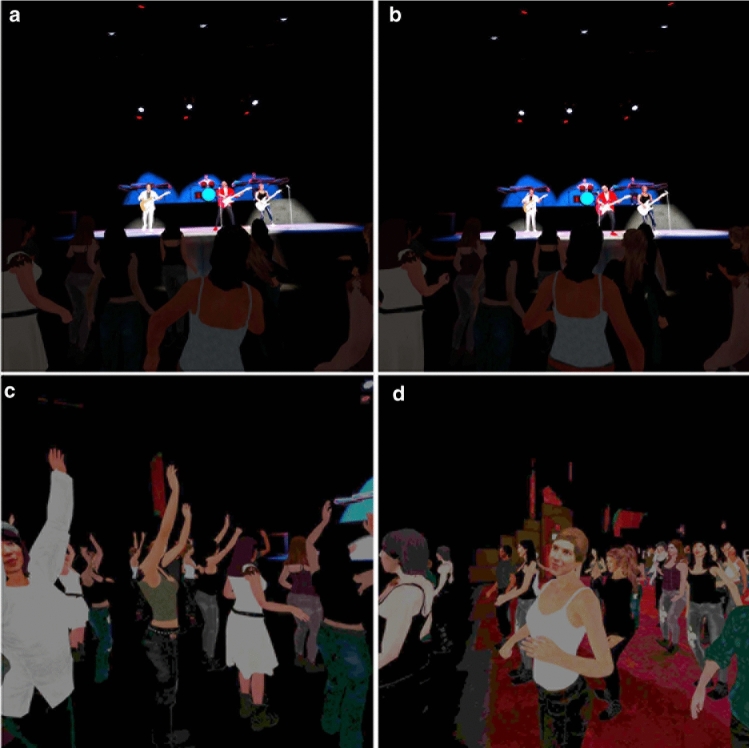
Images of the scenario (**a**)–(**b**) the band playing. **c** The audience is dancing along with the rhythm and one of the characters looks towards the participant. **d** The character to the right of the participant after looking at the participant

The VR scenario was a reproduction of the complete live performance of “Sultans of Swing” by Dire Straits which lasts just over 10 min. The participant was placed amongst a standing audience about 6 rows back from the stage. There was audience chatter to start and eventually an announcement welcoming Dire Straits. The band members ran onto the stage and took their positions, and then started to play. The crowd cheered and clapped, and did so again at various times during the performance, for example, clapping their hands above their heads, and cheering. Various aspects of the scenario are shown in Fig. 1. The video on https://youtu.be/2qdvNGjavEg shows the opening scenes.

### Procedures and ethics

Both studies were carried out during the COVID-19 pandemic so that face-to-face laboratory studies were not possible. Instead the Qualtrics software^1^ was used for both Studies 1 and 2. This is a survey tool accessed through a web interface. Participants were sent a link which opened to the information sheet, ethics consent form, instructions including installation of the software, demographic questions such as age, prior experience with VR, instructions on how to run the experience in the head-mounted display, and follow-up questions. Hence, participants could participate in their own time and place. When a participant had completed the experiment, the experimenters could access their responses from the Qualtrics pages. All responses were anonymous and IP addresses were not transmitted.

The studies were approved by the Bioethics Committee of the University of Barcelona, IRB00003099. All participants gave written and informed consent.

### Recruitment

Study 1 is described in detail in (Beacco et al. 2021). There were two groups of participants, 15 were recruited through an advertisement on social media, and 10 were students from a class in a United States University, which was not a technical computer science class. Of the 25 participants 17 identified as female and the remainder as male.

For Study 2, 17 were recruited from amongst students of various University classes (in Australia, New Zealand, and the United States), and a further 9 from advertisements on social media. 19 identified as male and 7 as female amongst those who finally participated. 65 individuals started the Qualtrics survey and 26 completed it. All of those who did not complete it stopped at the point that they were asked to download the application and upload it to their head-mounted display. Full demographic information for Studies 1 and 2 is shown in Supplementary Table S1. Participants were not paid for their participation.

### Equipment

All 25 of the participants in Study 1 used the Oculus Quest 1 head-mounted display supplied by their University. For Study 2 participants used either an Oculus Quest 1 (5), an Oculus Quest 2 (16), or the Pico NEO (5). The breakdown is given in Supplementary Table S1.

### Experimental design

For Study 1 all participants experienced the same environment, as described earlier. After the VR experience participants returned to the Qualtrics page, and answered the question labelled as “Essay” in Table 1.

**Table 1:** Post-experience questions

Variable name	Question
Essay	Please now write your answer below paying attention to the following points: The feeling to be at a concert Your movements along with the crowd (e.g. dancing, clapping, cheering) Aspects that drew you into the experience Aspects that drew you out of the experience Anything else you want to comment on (It would be helpful if your answer could be at least 150 words excluding copying the phrases above)
	After completing the essay question the Qualtrics moved to a separate block with 3 questions each on a 1–7 Likert scale, 1 = “Not at all”, 4 = “Sometimes”, 7 = “Almost all the time”
Copresence	How much, if at all, did you feel to be amongst a crowd of people?
Dancing	How much, if at all, were you swaying or dancing along with the audience?
Concert	Thinking back on your experience how much was this like attending a concert?

For Study 2 there was one binary factor that we refer to as Gaze with levels (NoLookAt = 0, *n* = 15) and (LookAt = 1, *n* = 11). Participants were allocated to these randomly by Qualtrics (across all 65 initial respondents). For those in the LookAt condition if they looked towards a nearby audience member the corresponding avatar had a high probability of returning their gaze with a smiling face. The return look lasted 1–3 s at random. For those in the NoLookAt condition the gaze behaviour of the participant had no effect on the surrounding virtual audience members. After the VR experience participants continued with the Qualtrics, and the questions are shown in Table 1.

### Implementation

Details of the major aspects of the implementation can be found in (Beacco et al. 2021). The following changes were made for Study 2:

- Improved colouring of the crowd—i.e. of the clothing
- Crowd animations were replaced by new mocap recordings that were properly synchronized with the music.
- Animations of the band members improved with a broader set of motions. We avoided penetration of guitars with skin meshes by using physics.
- Close crowd behaviour: when staring at some agents for a certain short number of seconds, they would look back at the participant for between 1 and 3 s.
- Added emulated dynamic lighting. By having two different baked lightmaps with different configurations (lights turned on and lights turned off), we could swap between them to simulate changes in the environment lightning.
- Random facial micro gestures on close crowd characters to give a more natural and organic look.

### Sentiment analysis

We used 4 different sentiment analysis methods available through R. Different systems use different dictionaries and various criteria. For example, the R package *sentimentr* (Rinker 2021) uses 9 dictionaries and aims particularly at “valence shifters” i.e. modifiers where “I do not like it” is correctly recognized as negative and “I really like it” is an enhanced positive valence. Rinker (2021)^2^ includes a comparative evaluation of several sentiment analysis packages.

*The VADER system* (Hutto and Gilbert 2014) was designed for the analysis of social media text but also is used more generally. Here we use the R implementation^3^ by Katherine Roehrick.

*The syuzhet package* (Jockers 2017)^4^ includes 4 sentiment lexicons and was originally designed for analysis of the latent structure in narrative, although it has been used widely for other applications.

*SentimentAnalysis*^5^ is the fourth R package that we use (Feuerriegel and Proellochs 2019; Feuerriegel et al. 2018) which exploits 3 different dictionaries which has applications in the analysis of financial text, but again has been used more widely.

There are many other sentiment analysis packages available, and comparisons are discussed in (Naldi 2019; Yoon et al. 2017). Rather than choose one particular package or complete an analysis using all of them and compare results, here we obtain results from all 4 packages mentioned above and treat them together. We obtain an $n\times 4$ matrix, where $n$ is the number of texts and the 4 columns are the sentiment scores for the 4 packages. Then cluster analysis is used to find subsets of the texts that have similar scores. Keywords and commonalities within each subset can be obtained, in order to identify the major themes that emerge in response to the concert experience.

We first consider only the texts produced in Study 2, and consider them per individual participant ($$n=26)$$. In this way we can also compare the sentiment analysis scores with the questionnaire responses. We then move on to analyse the combined results of Study 1 and Study 2 at the sentence level ($$n=611$$ sentences).

### Data availability

All data and programs for analysis are available on https://www.kaggle.com/melslater/analysis-of-concert-data.

## Results

### Observations from study 2

The overall impression from reading the essays written by participants in Study 2 is that once again their evaluations of the scenario were largely based on their responses to the audience. Those in the Gaze-LookAt condition were bothered by the virtual audience members staring back whenever they looked towards them, for example:

“… whenever I turned around the people closest to me stopped watching the concert and turned their attention toward me. I may have been imagining it, but it was none the less, a little creepy”.

“When I turned in the direction of someone they’d stare at me until I turned back, which was a little unsettling”.

“Every time I looked at the woman to my right and the woman to my left a little behind me, they turn to look at me and stare for the amount of time I would expect a friend to stare. Because they are strangers to me, I would not expect them to acknowledge my glances or to only look over with their eyes for a second. Turning their bodies and looking at me for such a long time made me feel uncomfortable”.

A second issue was that the audience consisted only of women:

“It was a bit weird that I was the only guy among an all women audience. Made it feel a bit like some sort of fantasy experience”.

“I personally felt a bit paralyzed watching the other concertgoers, most if not all who were beautiful women”.

“Furthermore, I felt very out of place as the only man between exclusively women”.

“Furthermore, it was very strange to be in an audience with all women. At the concerts I find myself going to, I am usually one of the very few women that makes it this close to the front of the stage”.

A third major issue was failure of expectation, which of course was also associated with the fact of there being a mainly female audience. Other examples include:

“The clapping did not sound right to me. It sounded as if it were also coming out of the guitar amps in the venue like the music. I would have expected it to sound much louder and closer to me”.

“The crowd stood farther apart than expected”.

“At no time did I really have the feeling of being at a real concert. Starting with the band, I missed the interaction with the audience as well as within the band itself”.

“The crowd seemed too far apart - like it was a socially distanced gig, which drew me out”.

The fourth major issue was simply technical issues or glitches:

“The models [of the band] also seemed to slide in a strange way when they walked sideways”.

“Overall it was a very entertaining experience, although it was clear that it is not a real concert, due to repetitive models, scaling issues, clashes of players on stage”.

“The restricted movement and graphics of the musicians and audience drew me out of the experience”.

“… the band was not actually playing in time to the music in a realistic way. The drummer was not playing on beat. The guitarist’s fingers were moving more like a bass player than like a guitarist”.

“They did not seem to move much and their movements seemed clunky”.

“The lead singer’s arm was clipping through his jacket sleeve, which seriously disrupted the immersiveness of the experience”.

On the positive side the spatialized sound was frequently mentioned as an important aspect of the experience.

“The spatial sound helped make it feel more real as well, along with crowd movements and noises”.

“The sound was quite good. As I moved my head around it felt like it was localised to the stage so that helped me feel more like I was there”. “Directionally, it felt like the sound was coming from in front of me and I was in the audience, which gave me an experiential concert feel and got me clapping along with the virtual audience”.

Also it is noteworthy that exactly the same scenario was experienced quite differently by different participants. For example,

“While the genre of music hits my taste, I found the experience unconvincing, lacking creativity, emotionless and unengaging”.

“I felt like I was at an unfulfilling virtual replica of a concert”.

On the other hand:

“This was a really cool experience! I immediately started dancing as if I were at the performance”.

“Overall, it was a very nice and interesting experience. I really felt that I am there in some way flexing with the musicians and the crowd. I was really about to clap at the end! And it was great to be at least at some kind of concert after several years of covid”.

### Sentiment analysis for study 2

Sentiment analyses were carried out for the complete texts of each the 26 participants. For the 4 packages positive sentiment scores indicate positive sentiment, negative scores negative sentiment, and scores around 0 indicate no sentiment expressed.

**Fig. 2:**
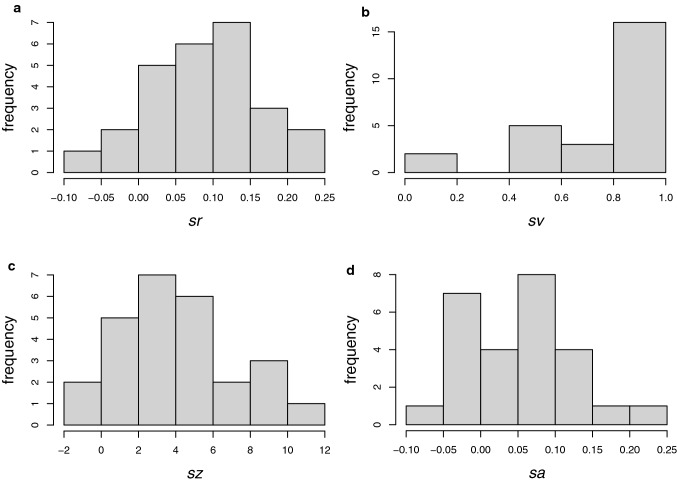
Histograms of sentiment scores for the 4 methods of Table 2 for Study 2. **a** sentimentr. **b** Vader. **b** syuzhet. **d** SentimentAnalysis

Table 2 shows the range, means and standard deviations of the 4 sentiment scores, and Fig. 2 the distributions. The vader method stands out as having a different distribution from the other 3. Table 3 shows the correlations between the scores of the different methods. While the correlation between sentimentr ($sr$) and vader ($sv$) is low, the others are positively correlated. Low correlation is useful since it means that the different variables are not responding to the same textual properties in the same way.

**Table 2:** Statistics for the Sentiment Scores for Study 2 by Sentiment Method (*n* = 26)

Package	VarName	Min	Max	Mean	SD
Sentimentr	$sr$	− 0.059	0.232	0.088	0.074
Vader	$sv$	0.064	0.993	0.764	0.260
Syuzhet	$sz$	− 1.950	10.250	4.025	3.050
SentimentAnalysis	$sa$	− 0.097	0.219	0.052	0.075

**Table 3:** Pearson correlations between the sentiment scores (with significance levels), *n* = 26

	$sv$	$sz$	$sa$
$sr$	0.27 (0.187)	0.51 (0.008)	0.43 (0.027)
$sv$	1	0.41 (0.036)	0.46 (0.018)
$sz$		1	0.53 (0.005)

**Fig. 3:**
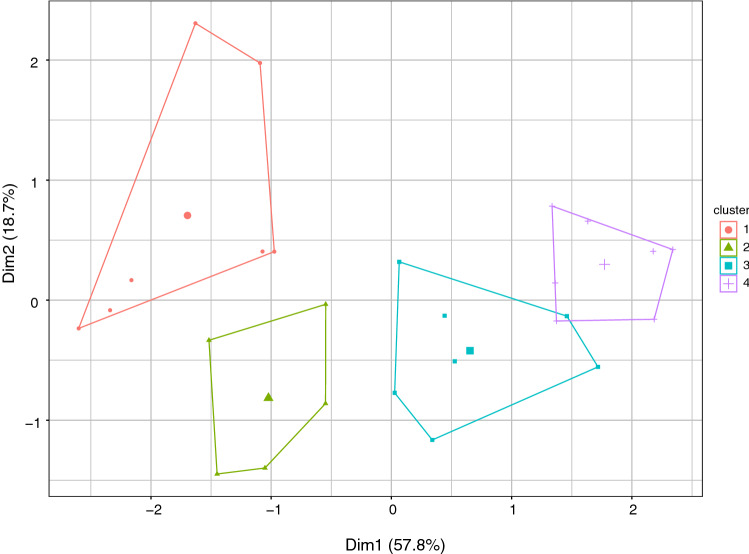
The first two principal components (Dim1 and Dim2) of the $26\times 4$ matrix of sentiment scores with the clusters shown by the convex hulls of their corresponding points. The clusters contain 7, 5, 7 and 7 in the order cluster 1 to cluster 4, respectively

Kmeans clustering (using kmeans in R) was used to cluster the resulting scores. 4 clusters resulted in the greatest separation between the clusters, more than 4 resulted in considerable overlap. An elegant way to show the clusters is to find the principal components (PCs) of the 4 sentiment scores, and plot the scores per individual of the first two PCs. The R package factoextra^6^ (Kassambara 2017; Kassambara and Mundt 2017) includes the function fviz_cluster that achieves this. Figure 3 shows the clusters plotted on the first two PCs. The first PC accounts for 58% of the total variance and the second 19%.

Table 4 shows the loadings for the PCs. The first PC is approximately proportional to the sum of the 4 sentiment scores (all loadings are 0.5 to one decimal place). ${PC}_{2}$ is proportional to the difference between the sentimentr score and the vader score. ${PC}_{1}$ and ${PC}_{2}$ account for almost 79% of the total variance, and we do not consider the remaining PCs further.

**Table 4:** Loadings for the principal components.

	${PC}_{1}$(58%)	${PC}_{2}$(19%)	${PC}_{3}$(12%)	${PC}_{4}$(11%)
$sr$	0.47	0.64	− 0.46	0.39
$sv$	0.45	− 0.74	− 0.50	0.08
$sz$	0.54	0.17	0.10	− 0.82
$sa$	0.53	− 0.12	0.73	0.41

The $j=\mathrm{1,2},\mathrm{3,4}$ PC score for individual $i=\mathrm{1,2},\dots ,n\left(=26\right)$ is the row vector $({sr}_{i},{sv}_{i},{sz}_{i},{sa}_{i})$ multiplied by the column vector ${PC}_{j}$. The brackets show the proportion of variance explained by the corresponding PC

**Fig. 4:**
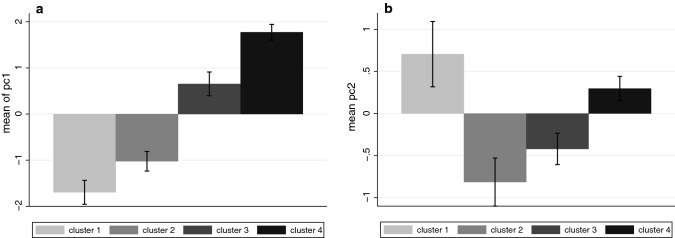
Mean and standard errors of the PC scores by the clusters (**a**) ${PC}_{1}$ (**b**) ${PC}_{2}$

Table 5 shows the correlations between the PCs and the sentiment variables. It can be seen that ${PC}_{1}$ is strongly positively correlated with all 4 variables. ${PC}_{2}$ is strongly positively correlated with $sr$, negatively with $sv$ and not with the remaining two PCs. Figure 4 shows the means and standard errors of the PC scores by the clusters.

**Table 5:** Correlations between the first two principal components and the sentiment scores (significance levels)

	$sr$	$sv$	$sz$	$sa$
${PC}_{1}$	0.72 (0.00003)	0.69 (0.0001)	0.82 (0.0000003)	0.81 (0.0000006)
${PC}_{2}$	0.55 (0.003)	− 0.64 (0.0005)	0.15 (0.5)	− 0.11 (0.6)

Using this information, we can interpret Fig. 3. The first dimension ${PC}_{1}$ is positively associated with all sentiment scores. Clusters 2, 3 and 4 are in the order of increasing levels of sentiment on the first dimension. Cluster 1, however, is low on the first dimension and higher on the second dimension. This indicates ambivalence in the responses: “one the one hand …[something positive]”, and “on the other hand … [something negative]”.

We can summarize the text associated with the 4 clusters using a keyword extraction technique and sentence summaries. We use the udpipe package (Straka et al. 2016; Straka and Straková 2017)^7^ and in particular dependency parsing, which finds nominal subjects and the adjectives that describe them. See also.^8^ We use the lexRank (Erkan and Radev 2004)^9^ method to summarize the text in the clusters. The results are shown in Table 6.

**Table 6:** Keyword pairs, the context of those keyworks, and summary

Keyword pairs	Context	Summary
*Cluster 1*		
Effective one	As far as virtual experiences go, this one was pretty effective at reproducing the concert experience	[1] "Part of the magic of a concert experience is the collective effervescence of the crowd that the music draws and I felt that this was missing from the experience" [2] "Knowing that this was a Dire Straits concert, I would assume that the crowd would be more diverse" [3] "As a female one of my favorite things about going to a concert is looking around at the crowd during the show and taking fashion inspiration from the women around me" [4] "The music and crowd motion drew me into the experience" [5] "At the concerts I find myself going to, I am usually one of the very few women that makes it this close to the front of the stage"
Weird drummer	The drummer and pianist running across the stage while hunched over was a bit weird as well, but did not impact my overall experience to a large degree	
Evident concert	The environment was well designed and the feeling of being at a real concert was evident during certain moments	
Long song	I felt that I was really at the concert. However I am not a huge fan of Dire Straits so the song was very long	
Diverse crowd	Knowing that this was a Dire Straits concert, I would assume that the crowd would be more diverse	
Female audience	I was drawn out of the presence by the fact that the audience was all female and not very varied in race and ethnicity	
Generic clothing	I found that the avatars clothing and style was quite generic and modest in style in comparison to what I would typically see at a show	
*Cluster 2*		
Female audience	The audience was all female, and they seemed to be looking at me a lot of the time, also disconcerting Interestingly, it took me until about halfway through the song to realize that the entire audience was female	[1] "Sometimes I decided to move a bit along with the crowd, dancing, waving arms.. " [2] "Avatars would start clapping just a little bit after the audio of the crowd started cheering, which as pretty eerie, and when the crowd in the audio was clapping to the beat, avatars were clapping off of the beat, which destroyed that cool synchronous collective feeling you get being in a crowd at a concert" [3] "The crowd was mostly realistic, but the things that broke immersion were the timing on clapping and the limited number of avatars" [4] "At times it felt a bit like being part of a crowd" [5] "I did sometimes have the feeling of being at a concert, especially when the crowd started cheering, but I am doing this in the afternoon, while I am a little tired and not exactly in a concert-going mood"
Female crowd	The crowd stood farther apart than expected. It was unusual that the crowd was only female	
High motivation	The motivation to dance and clap along was rather high	
Disconcerting performer	It was hard to feel immersed in the experience, the music felt real, but the avatar performers and audience were more disconcerting than immersive	
Realistic crowd	The crowd was mostly realistic, but the things that broke immersion were the timing on clapping and the limited number of avatars	
Repetitive movement	Movements were repetitive and animations imprecise	
*Cluster 3*		
Great sound	It definitely felt like I was at a concert, the sounds were great at drawing me into the experience The sound traveling from one ear to the other when I changed direction was great	[1] "I wouldn’t say that it felt like I was alone but I would much prefer to be completely alone without any of the audience in that concert" [2] "It definitely felt like I was at a concert, the sounds were great at drawing me into the experience" [3] "The audiences near me on the other hand are very disturbing" [4] "The timing of screaming and clapping sound of the audiences did not match with their animation either" [5] "I noticed myself swaying along with the crowd at some points during the concert"
Nice music	The live music was nice	
Effective audience	The audience’s cheering was also effective	
Good effect	No spatial audio effect is not good for the VR experience I think	
Mandatory audio	Spatial audio should be mandatory for this kind of application since people can move their bodies and head around	
Immersive sound	Upon beginning the experience, I noticed two things: the sounds being very immersive, but the human models taking away from some of this feeling of immersion	
Believable movement	On the other hand, the movements of the crowd were mostly believable	
Great direction	The sound traveling from one ear to the other when I changed direction was great	
Slight sense	The sense of being at concert was slight	
Impactful audio	In particular, the high fidelity audio of a live recording was impactful in that way [the sense of being at a concert]	
Distract use	The guys on stage didn’t look realistic and that pulled me out. Their use of the instruments was distracting	
Weird thumb	Didn’t look like they were playing guitar and the thumbs especially were weird	
Ok clapping	The clapping in the audience was ok if you didn’t look too close	
Fun seconds	First few seconds was fun. Hearing the band starting the song and addressing the audience, hearing and seeing the audience response and move for the first time are really exciting	
Gone immersion	[continuation of previous entry] But as soon as I looked around, my immersion was instantly gone because the audiences around me just stopped reacting to the music and looked at me instead	
Strange animation	Some audiences animations were really strange	
Decent venue	The venue and lighting were decent in my opinion	
Disturbing audience	The audiences near me on the other hand are very disturbing	
*Cluster 4*		
Irresistible movement	When the audience put their hands in the air I also felt compelled to do the same. The movements were quite irresistible	[1] "So it didn’t feel close to real in that sense but it still felt like I experience something" [2] "Not having hands made me feel a bit less in the experience" [3] "Made it feel a bit like some sort of fantasy experience" [4] "Having the crowd was also fun as I felt that I somehow have shared the experience with others" [5] "The crowd moved a lot more naturally than a lot of other NPC-based experiences so that helped me get in to the groove more"
Good sound	The sound was quite good. As I moved my head around it felt like it was localised to the stage so that helped me feel more like I was there	
Excellent quality	One thing I will say is that the recorded sound quality of the performance was excellent and I felt in the Quest 2 headset that it did simulate a live sound experience	
Stationary hand	They should have been moving their arms as if they had a pick in their hand. Both the guitarists and bassist’s left hand were mostly stationary, while they should have been sliding up and down the neck of their instruments according what notes they were playing at a given time	
Quantifiable programmer	Again, I am not a computer programmer at all, but a lot of musical data would be easily digitized (stuff like tempo, meter, pitch, etc. is all very quantifiable) and if you could get an application like Logic/Protools/Garageband to play the song in midi, perhaps it would be possible to send that midi-data into the animation software to get the Avatars to move in time with the song?	
Enough stage	The stage was not large enough and could have been better dressed with front monitors and lighting, amps, steps, etc	
Female crowd	The crowd were exclusively female, which would be highly unlikely for a Dire Straits concert – and none of them were drinking (also, if this was in the 1980s, people would have been smoking)	
Friendly face	However, there were a girl on the right of me that was literally staring at me when I looked at her. And her face was not friendly at all. At some point she even started to move closer and dancing in a very uncomfortable proximity from me But having an eye-contact with a third girl who had a really friendly face was super nice!	
Nice move	And the clapping thing of the audience as well as some synchronized moves was nice and inviting	
Distracting creeps	The creeps from the audience and the standing-too-close girl were very distracting	
Important audience	But the audience was sometimes more important that the band itself in this experience"	
Unnerving fact	Also, the fact that they turn to look at me every time I glance at them was unnerving	

Each cluster contains a mix of positive and negative sentiment, following Fig. 3. For the clusters 2, 3, 4 even though they show increasing sentiment for ${PC}_{1}$ they are generally on the low side of ${PC}_{2}$. However, from 2 to 4 the proportion of positive statements increases. Cluster 1 is low on ${PC}_{1}$ indicating that it is low across all 4 sentiment scores, whereas it is high on ${PC}_{2}$ indicating a low score on vader. Correspondingly most of the comments show negative sentiment.

An important point throughout is that overwhelmingly negative comments relate to the audience not to the band itself:

- Lack of diversity
- Incorrect clothing
- Lack of synchrony between sounds and observed movements of the audience
- Audience members looking at the participant inappropriately.

On the other hand the audience was also effective in pulling people into the experience, so that they would find themselves dancing along with the audience.

Sound was important in generating positive sentiment, in particular the spatialized sound (though one participant did not perceive this). With respect to the band, incorrect movement while playing the guitar generated negative sentiment.

### Analysis of the questionnaire scores of study 2

**Fig. 5:**
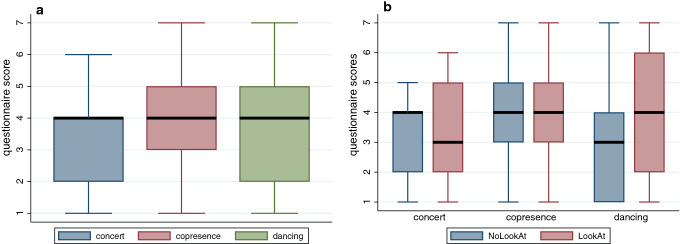
Box plots of the questionnaire responses (**a**) Overall. **b** By the Gaze condition. The thick horizontal lines are the medians, the boxes are the interquartile ranges (IQR), the whiskers extend from max(min value, lower quartile − 1.5*IQR) to min(max value, upper quartile + 1.5*IQR)

Here we consider the scores for the questions shown in Table 1. In particular we were interested in whether the Gaze condition had any influence on the variables concert, copresence, or participants dancing along with the audience (dancing). Figure 5 shows the box plots. Overall the levels of response were low in the case of concert, and approximately symmetrically distributed around the median in the other two cases. In terms of the effect of the Gaze condition there appears to be no or little difference between the NoLookAt and LookAt conditions (formal analysis confirms this).

Next we consider whether there is any relationship between the sentiment scores and the questionnaire variables. The three questionnaire response variables are highly correlated. Spearman’s rho between concert and copresence is $\rho =0.66$ (*P* = 0.002), between concert and dancing $\rho =0.57$ (*P* = 0.0025) and between copresence and dancing $\rho =0.50$ (*P* = 0.009). The questionnaire score concert is highly correlated with one of the sentiment scores $sr$ ($\rho =0.53$, *P* = 0.006) but not with any of the others. The variable concert is also positively correlated with ${PC}_{2}$ ($\rho =0.47$ (*P* = 0.016), but not with ${PC}_{1}$. There is also an influence of gender.

**Fig. 6:**
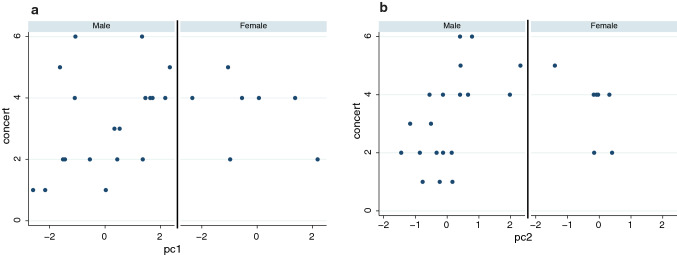
Scatter plot of concert on PCs by gender (**a**) ${PC}_{1}$ (**b**) ${PC}_{2}$

Figure 6 shows the scatter plots of concert on the PCs by gender. Figure 6a suggests that for ${PC}_{1}$ for males there is a positive correlation between concert and sentiment, whereas for females negative. The same is shown for ${PC}_{2}$ in Fig. 6b. (The same results can be found if $sr$ is used instead of the PCs). The interpretation is that for males there is a positive association between being at the concert and positive sentiment. However, for females, a high sense of being at the concert is associated with lower sentiment. As noted in the analysis above the feeling of being stared at, for example, causing negative sentiment, only makes sense if there is an illusion of actually being at the concert, i.e. the events are interpreted as if really occurring. (There are very similar graphs for copresence and to a lesser extent for dancing). The result indicates a difference in response between males and females.

For almost all our studies over the past 7 years we have used Bayesian statistical analysis. A particular advantage is that multiple response variables can be considered simultaneously in one overall model, affording multiple inferences across the various models. In comparison, in classical statistics, more than one significance test results in problematic interpretation of significance, and ad hoc methods are required to try to overcome this problem. A Bayesian logistic regression was therefore carried out for each of the three questionnaire response variables concert, copresence, and dancing, in one overall model that includes all three. Logistic regression is used because the response variables are ordinal. For each, the linear predictor is of the form:

$${\text{Gender }} + {\text{ }}PC1{\text{ }} + {\text{ }}PC1 \times {\text{Gende}}r{\text{ }} + {\text{ }}PC2{\text{ }} + {\text{ }}PC2 \times {\text{Gender }} + {\text{ HMD}}1{\text{ }} + {\text{ HMD}}2$$

where Gender (Male = 0, Female = 1) is the main effect of gender, P*C1 *is the main effect of ${PC}_{1}$, and* PC*1 × Gender is the interaction term, and similarly for P*C2*. HMD1 corresponds to Quest 1, HMD2 to Quest 2, where these are binary variables equal to 1 for the corresponding HMD and 0 otherwise. Hence the Pico corresponds to HMD1 = HMD2 = 0. Formally the linear predictor of the model for the $i$ th individual is:

$$\beta _{0} + \beta _{1} {\text{gender}}_{i} + \beta _{2} PC_{{1,i}} + \beta _{3} \left( {{\text{gender}}_{i} \cdot PC_{{1i}} } \right) + \beta _{4} PC_{{2i}} + \beta _{5} \left( {{\text{gender}}_{i} \cdot PC_{{2i}} } \right) + \beta _{6} {\text{HMD}}1_{i} + \beta _{7} {\text{HMD}}2_{i}$$

where the ${\beta }_{j}$ are replaced by the coefficients indicated in the first column of Table 7 for the three different response variables. The prior distribution for each coefficient was taken as normal (mean = 0, SD = 10), so that the prior 95% credible intervals are − 20 to 20. The logistic model also requires cut points, and the prior distributions were similarly assigned normal (mean = 0, SD = 10). These are weakly informative prior distributions (Gelman et al. 2008; Lemoine 2019), meaning that they are proper probability distributions, but with high variance representing high uncertainty.

**Table 7:** Summaries of the posterior distributions showing for each parameter the mean of the distribution, the standard deviation, the 95% credible interval

Parameter	Coefficient of:	Mean	SD	2.5%	97.5%	Prob > 0
Concert						
${\beta }_{con,0}$		− 2.59	4.09	− 10.94	5.26	0.262
${\beta }_{con,1}$	Gender	0.80	1.02	− 1.12	2.81	0.779
${\beta }_{con,2}$	*PC*1	1.07	0.39	0.34	1.87	0.998
${\beta }_{con,3}$	*PC*1 × Gender	− 1.53	0.71	− 2.97	− 0.17	0.013
${\beta }_{con,4}$	*PC*2	1.88	0.64	0.69	3.17	0.999
${\beta }_{con,5}$	*PC*2 × Gender	− 3.79	1.78	− 7.29	− 0.27	0.019
${\beta }_{con,6}$	HMD1	0.56	1.40	− 2.17	3.31	0.654
${\beta }_{con,7}$	HMD2	− 0.08	1.06	− 2.14	2.01	0.471
Copresence						
${\beta }_{cop,0}$		− 0.98	2.94	− 6.76	4.81	0.371
${\beta }_{cop,1}$	Gender	1.17	0.94	− 0.66	3.06	0.901
${\beta }_{cop,2}$	*PC*1	0.45	0.34	− 0.20	1.15	0.909
${\beta }_{cop,3}$	*PC*1 × Gender	− 0.73	0.61	− 1.93	0.50	0.111
${\beta }_{cop,4}$	*PC*2	0.81	0.50	-0.14	1.82	0.950
${\beta }_{cop,5}$	*PC*2 × Gender	1.31	1.65	− 1.85	4.68	0.781
${\beta }_{cop,6}$	HMD1	2.11	1.58	− 0.98	5.31	0.913
${\beta }_{cop,7}$	HMD2	0.87	1.16	− 1.45	3.16	0.779
Dancing						
${\beta }_{dan,0}$		− 0.11	9.87	− 19.33	18.84	0.495
${\beta }_{dan,1}$	Gender	1.62	0.92	− 0.18	3.48	0.961
${\beta }_{dan,2}$	*PC*1	0.43	0.35	− 0.26	1.11	0.892
${\beta }_{dan,3}$	*PC*1 × Gender	− 0.85	0.62	− 2.08	0.38	0.089
${\beta }_{dan,4}$	*PC*2	− 0.14	0.59	− 1.31	1.03	0.412
${\beta }_{dan,5}$	*PC*2 × Gender	2.13	1.82	− 1.32	5.85	0.887
${\beta }_{dan,6}$	HMD1	5.85	2.05	2.11	10.16	1.000
${\beta }_{dan,7}$	HMD2	3.48	1.39	0.91	6.36	0.996

Prob > 0 is the posterior probability that the parameter > 0

The results are summarized in Table 7.

Regarding the concert response variable, the evidence is very strong that higher values of* PC*1 or* PC*2 are associated with lower values in the case of females, and higher values in the case of males (i.e. when Gender = 0). In the case of copresence,* PC*1 has a negative association in the case of females, and positive for males. There is no interaction of gender with* PC*2, but* PC*2 is positively associated with copresence independently of gender.

In the case of dancing* PC*1 is associated with lower scores for females but* PC*2 with higher scores. Also* PC*1 and* PC*2 are positively associated with all three responses irrespective of gender. From analysis of the questionnaire data there is no evidence that the Gaze condition influenced these scores, but there is quite strong evidence of an association with the sentiment scores, moderated by gender. There is also some effect of the type of HMD. For copresence the Quest 1 is associated with higher values than the Pico. For dancing both Quest 1 and Quest 2 are associated with greater values than the Pico. Considering the overlaps between the credible intervals for Quest 1 and Quest 2 there appears to be no difference between these two.

These results should be treated with caution because there are only 7 out of 26 females. However, the Bayesian method clearly updated the high variance prior distributions of the parameters, indicated by the narrow posterior 95% credible intervals (compared with the prior intervals of − 20 to 20). Thus these data were sufficient to move from a probability model with very wide variances to one with low variances.

### Analysis at the sentence level (studies 1 and 2 combined)

Above we only analysed the results of Study 2, treating the entire essay written by each person as the text for the sentiment score. As we have seen, each essay may contain both positive and negative sentiment, and the single overall score will reflect that with positive and negative sentiment cancelling each other. Here we treat all the sentences of Study 1 and Study 2 together, and create sentiment scores at the sentence level. There are *n* = 611 sentences altogether. We follow a similar strategy of obtaining scores on the same variables $sr$, $sv$, $sz$ and $sa$, and then find clusters amongst these with the help of the principal components.

**Fig. 7:**
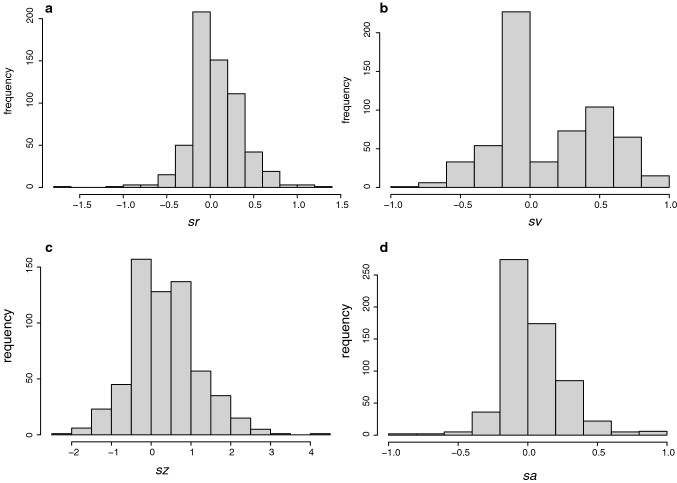
Histograms of sentiment scores for the 4 methods of Table 8 for all sentences combined. **a** sentimentr. **b** Vader. **c** syuzhet. **d** SentimentAnalysis

Table 8 shows the statistics for the 4 methods, and Fig. 7 the corresponding histograms. The vader method ($sv$) stands out as a different distribution. Table 9 shows that all scores are highly positively correlated with each other.

**Table 8:** Statistics for the Sentiment Scores for all sentences by Sentiment Method (*n* = 611)

Package	VarName	Min	Max	Mean	SD
Sentimentr	$sr$	− 1.736	1.292	0.083	0.293
Vader	$sv$	− 0.818	0.947	0.157	0.357
Syuzhet	$sz$	− 2.350	4.350	0.425	0.813
SentimentAnalysis	$sa$	− 1.000	1.000	0.073	0.218

**Table 9:** Pearson correlations between the sentiment scores over all sentences, *n* = 611

	$sv$	$sz$	$sa$
$sr$	0.509	0.579	0.492
$sv$	1	0.608	0.479
$sz$		1	0.505

All significance levels are 0

Next we find the principal components with the loadings shown in Table 10. Note that ${PC}_{1}$ is reversed compared to the sentiment scores (i.e. higher values correspond to lower sentiment), and is approximately proportional to the (negative) sum of the 4 sentiment scores. ${PC}_{2}$ is approximately $sv+sz-sa$. Since the first two PCs account for 79% of the variance we do not consider the remaining two.

**Table 10:** Loadings for the principal components

	${PC}_{1}$(65%)	${PC}_{2}$(14%)	${PC}_{3}$(12%)	${PC}_{4}$(9%)
$sr$	− 0.50	0.06	− 0.80	− 0.32
$sv$	− 0.50	0.42	0.55	− 0.52
$sz$	− 0.52	0.31	0.03	0.79
$sa$	− 0.47	− 0.85	0.22	0.01

The $j=\mathrm{1,2},\mathrm{3,4}$* PC *score for sentences $i=\mathrm{1,2},\dots ,n\left(=611\right)$ is the row vector $({sr}_{i},{sv}_{i},{sz}_{i},{sa}_{i})$ multiplied by the column vector ${PC}_{j}$. The brackets show the proportion of variance explained by the corresponding* PC*

Table 11 shows the correlations between the* PC*s and the sentiment variables. ${PC}_{1}$ is highly negatively correlated with all scores, and ${PC}_{2}$ is positively correlated with $sv$ and $sz$ and negatively with $sa$.

**Table 11:** Correlations between the first two principal components and the sentiment scores over all sentences (*n* = 611)

	$sr$	$sv$	$sz$	$sa$
${PC}_{1}$	− 0.80	− 0.81	− 0.84	− 0.76
${PC}_{2}$	0.05	0.31	0.23	− 0.63

All significance levels are effectively 0 except between ${PC}_{2}$ and $sr$ which is 0.25

**Fig. 8:**
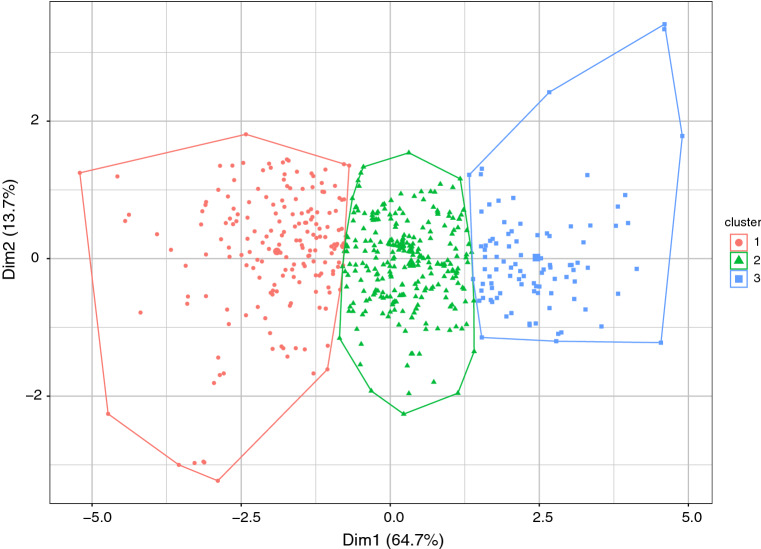
The first two principal components (Dim1 and Dim2) of the $611\times 4$ matrix of sentiment scores over all sentences with the clusters shown by the convex hulls of their corresponding points. The clusters contain 183, 334, and 94 in the order cluster 1 to cluster 3, respectively

The number of clusters keeping clear separation between them is 3 (more than this and the clusters overlap considerably). The clusters are shown in Fig. 8. Recall that the ordering inverted with 1 representing the highest sentiment and 3 the lowest. Table 12 shows the means and standard errors of the* PC*s. For ${PC}_{1}$ as expected there are clear differences between the means ranging from low to high in the order from 1 to 3. However, for ${PC}_{2}$ there is not much difference between the clusters, which is also clear from Fig. 8 since the clusters are not well distinguished on the second dimension. ${PC}_{1}$ being approximately the sum of the 4 sentiment scores (though with sign reversed) is the more useful dimension.

**Table 12:** Means and Standard Errors of the PCs by cluster

	Mean ${PC}_{1}$	SE ${PC}_{1}$	Mean ${PC}_{2}$	SE ${PC}_{2}$
Cluster 1	− 1.901	0.065	0.105	0.069
Cluster 2	0.352	0.031	− 0.062	0.031
Cluster 3	2.452	0.086	0.015	0.085

**Fig. 9:**
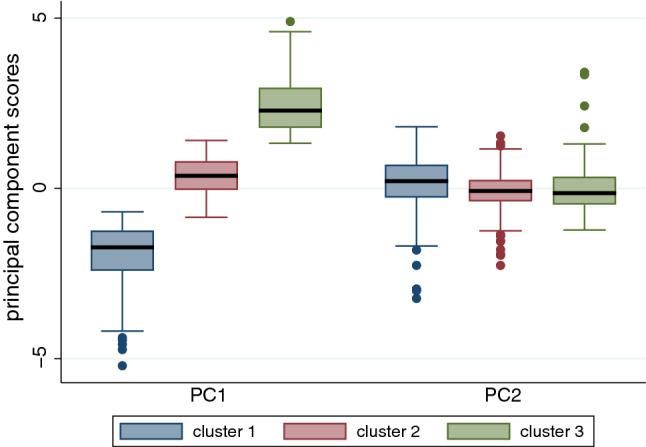
Boxplots of sentiment by cluster for ${PC}_{1}$ and ${PC}_{2}$

**Fig. 10:**
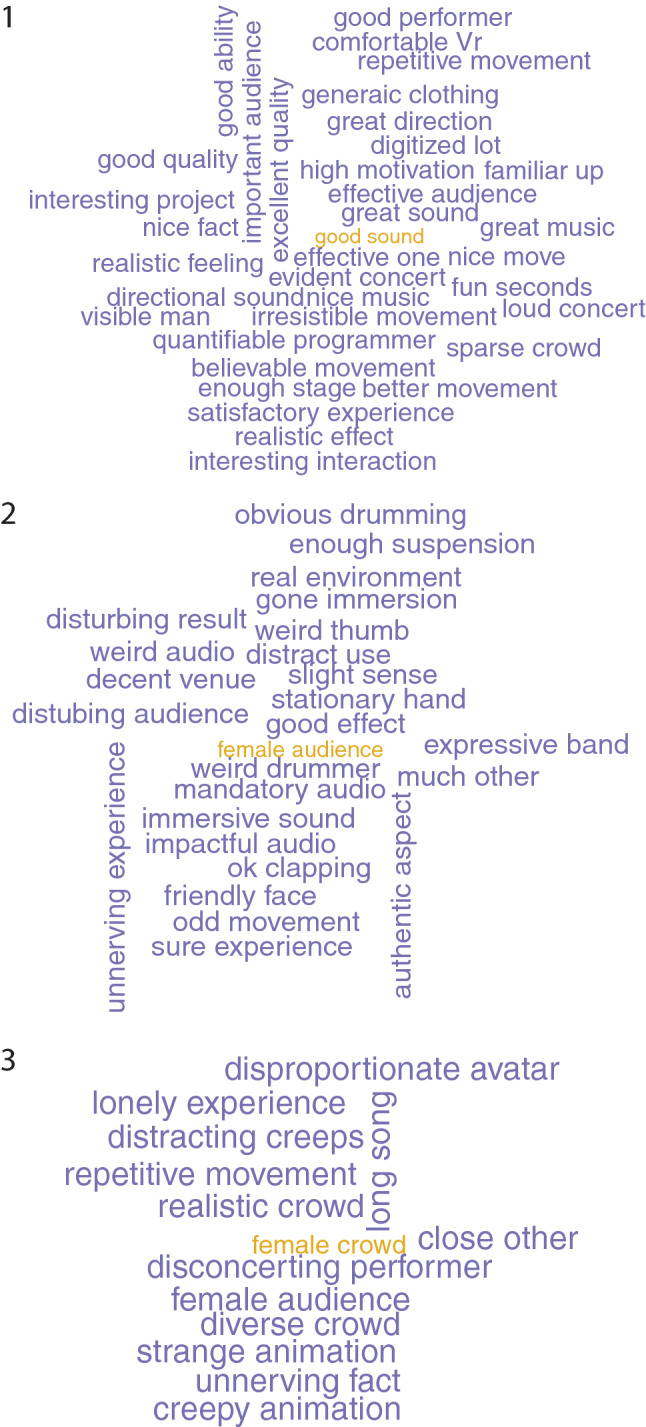
Word clouds of keyword pairs showing nominal subjects and the adjectives that describe them, by cluster. All frequencies are 1 except for those with the lighter colour

Figure 9 shows the clear separation in sentiment scores represented by ${PC}_{1}$ and that ${PC}_{2}$ has little variation across the clusters, though with more low value outliers in cluster 1, and more high value outliers in cluster 3. Figure 10 shows the keyword pairs, i.e. nominal subjects and their corresponding adjectives. The purpose of this is just to get a quick overview of the sentence types in the different clusters. It is clear that cluster 1 has the highest sentiments and 3 the lowest, with 2 in-between.

Table 13 shows the summary sentences for each cluster. Cluster 1 (with the highest sentiment) concentrates almost wholly on the positive impact of the sound and the audience behaviour. Where the band is mentioned (sentence [14]) it is the only negative aspect.

**Table 13:** Summaries of the text in the clusters using lexRank, showing the top 15 sentences in each case. Sentences are classified as either relevant to PI, or Psi factors (i), (ii), (iii). A—sign indicates a contribution to negative sentiment

Cluster 1 (*n* = 183)
[1] "The sound was good and felt like real live music at times". (Psi (iii)) [2] "It definitely felt like I was at a concert, the sounds were great at drawing me into the experience" [3] "I felt included in the concert crowd, watching them dancing would encourage me to move and dance as well but after a while, the movements were repetitive so I felt a bit more self-aware" [4] "I didn’t clap my hand because I just didn’t feel like in concert even though the sound quality and song sounded like in the actual concert" [5] "Having the crowd was also fun as I felt that I somehow have shared the experience with others" [6] "So it didn’t feel close to real in that sense but it still felt like I experience something" [7] "The spatial sound actually makes it feel like at an actual concert, the audiences really helps this experience to be very interactive". (PI) [8] "I enjoyed the movement around me, being surrounded by people dancing was good, although not realistic/appropriate for the performance or particularly in time with the music". (Psi -(iii)) [9] "The spatial sound helped make it feel more real as well, along with crowd movements and noises". (PI) [10] "It felt nice to be in a crowd of people dancing and I felt compelled to join in" [11] "The set up is so familiar—a crowd, darkness, a well lit stage, the music, the fact that if you turn your head, the sound is directional, and the identifiable members of the band—all drew me, making it feel more like a real experience". (PI, Psi (iii)) [12] "When the people around me started dancing I really felt like dancing as well, especially during the parts when they were all cheering and clapping" [13] "Lastly, towards the end of the performance, I clapped and made sounds (woohoo, etc.) but because I could not hear anyone else making sounds it felt weird: it would have been awesome if there was more noise from the crowd like a real concert". (Psi -(iii)) [14] "The band performance did not feel real so I wasn't so engaged with the music as I normally would do in a concert, but would be keen to see and actual live concert through VR.. " [15] "I enjoyed being able to move around the crowd"
*Cluster 2 (n = 334)*
[1] "I was feeling the need to move closer to the stage during the whole experience (as I would do in a real concert) ". (Psi –(iii)) [2] "I did not really feel like moving at all mainly because the band wasn't moving, the music was not loud enough, the people around me didn't move too much and also didn't move like the crowd in the back". (Psi -(iii)) [3] "Movements: I moved as if I was at a real concert" [4] "The crowd did not move the way people usually move in a concert and usually they sing along, there is always some crowd noise". (Psi -(iii)) [5] "The sound music, the lighting and the crowd really drew me into the experience" [6] "Feeling the crowd dancing around me drew me in" [7] "The band themselves drew me out a bit- they didn't move much while they played". (Psi -(iii)) [8] "The audience really drew me into the experience" [9] "Not having hands made me feel a bit less in the experience". (Psi -(iii)) [10] "The music and crowd motion drew me into the experience" [11] "Otherwise, I thought it was a very immersive experience and I felt like I was in a crowded concert!" [12] "At no time did I really have the feeling of being at a real concert" [13] "One aspect that drew me out was there were times in the audio that it sounded like the audience was clapping but when I looked around, the audience was not visually clapping". (Psi -(iii)) [14] "The whole illusion felt like a real concert venue- the lighting, the crowd (but with more room to move than in real life) " [15] "It was nice to be a part of concert but the movement of the crowd was a bit odd, some of the people only moved when I looked in their direction". (Psi -(iii))
*Cluster 3 (n = 94)*
[1] "It was hard to feel immersed in the experience, the music felt real, but the avatar performers and audience were more disconcerting than immersive the audience was all female, and they seemed to be looking at me a lot of the time, also disconcerting". (Psi –(ii) –(iii)) [2] "Two very creepy looking people on my right kept staring at me the whole time and that made me uncomfortable". (Psi –(ii)) [3] "At one point the avatar to my left made a strange arm movement that broke the illusion of presence". (Psi –(iii)) [4] "As negative, I have to say that everything seemed too static: starting from my inability to move, but also the fact that the crowd didn't move and that there were no groups amongst the crowd, just people dancing alone". (Psi –(iii)) [5] "The two people dancing on the left and right of me definitely drew me into the experience, but also made me quite uncomfortable" [6] "Turning their bodies and looking at me for such a long time made me feel uncomfortable". (Psi –(ii)) [7] "Furthermore, the quite static and unnatural movements of the band and the audience were always a point that continuously reminded me that this is not a real concert". (Psi –(iii)) [8] "Some audiences animations were really strange". (Psi –(iii)) [9] "In many moments when I turned my head or some other movement, the image of my virtual hands appeared that were staying in strange ways, that aspect disconnected me a bit from the experience and I had to move my arms so that the image of the hands disappeared". (Psi –(iii)) [10] "I noticed that all of the crowd seemed to be woman, all of 5–6 different avatar types which was a little strange". (Psi –(iii)) [11] "The crowd was mostly realistic, but the things that broke immersion were the timing on clapping and the limited number of avatars". (Psi –(iii)) [12] "The solid eye contact of the man on my right was a little creepy, and as a woman, made me feel uncomfortable". (Psi –(ii)) [13] "When I looked around the woman to my right seemed to hold my gaze, this felt weird, I guess it drew me in, or it made me feel uncomfortable, I then looked further around and another woman looked at me, just for too long to be normal". (PI, Psi –(i)) [14] "Furthermore, it was very strange to be in an audience with all women". (Psi –(iii)) [15] "The faces were also occupying a bit of the Uncanny Valley, which made me feel a little bit uncomfortable and took me out of the experience a bit"

Considering cluster 3 (with the lowest sentiment) three aspects emerge: the audience members staring at the participant and “creepy” eye contact, inappropriate movements of the crowd, the fact that the crowd was women only. Again almost all sentences refer to the crowd, with one referring to the band ([7]).

Cluster 2 shows a mixed set of responses, mentioning both some positive and negative aspects—the inability to move towards the stage, the movements and noises from the crowd not being appropriate, the lack of movement of the band, but the audience drawing them into the experience, and the venue, lighting and crowd drawing them into the experience.

The notations following some of the sentences approximately classify the meaning as corresponding to PI or the three factors contributing to Psi, with positive or negative sentiment. For example cluster 1 [11] references a sensorimotor contingency “… the fact that if you turn your head, the sound is directional …” which contributes to PI, and also “… the identifiable members of the band …” which conforms to expectation appropriate to a Dire Straits performance, Psi condition (iii). Cluster 3 [7] refers to “ … quite static and unnatural movements of the band and the audience …” referring also to a failure of expectations, resulting in negative sentiment.

## Discussion

The most informative experiments are often those where the results are unexpected since this is when we can learn something new, although of course hypothesis testing studies to evaluate specific theories or replicate past findings are also important. In Study 1 we were surprised to find how significant the appearance and behaviour of the audience were in shaping the responses of some participants. In Study 2 the same happened, even though we had aimed at removing the aspects of audience appearance that had caused distress for some participants (i.e. that the audience members appeared as male) and simulated some interaction with the audience through their returning participant glances. Our focus has been mainly on aspects that resulted in low sentiment, because this was the most unexpected and striking, and also is critical for the production of later improved versions of the VR scenario. As one participant wrote positively: “The sound was good and felt like real live music at times”. This is nice to know, but it is not too informative. On the other hand “… the quite static and unnatural movements of the band and the audience were always a point that continuously reminded me that this is not a real concert”. This points to an actual improvement that later versions of the scenario need to address.

Almost all of the negative sentiment issues in Table 13 summaries for cluster 3 are related to Plausibility. Recall the three components of Psi (i) responsiveness, (ii) contingent actions towards the participant and (iii) expectations. For example, Cluster 3 [1] refers to “the audience was all female” which is a failure of expectation, and “they seemed to be looking at me a lot of the time, also disconcerting”. This is the second aspect contributing to Psi, where from the standpoint of the participant contingent events occur that refer directly to the participant. From this statement is it not clear whether the “looking at” was in response to the participant looking at the character, in which case it would correspond to condition (i), or whether the participant thought that the character spontaneously looked. It is important to note that Psi does not necessarily result in positive sentiment. In this case a character looks at the participant (for too long) and this is experienced as uncomfortable. The very operation of Psi is itself what has resulted in negative sentiment. In fact the negative sentiment occurs because there is Psi, the illusion that the events are really happening—that someone was looking at the participant. If participants would not have the illusion of actually being stared at then there would be nothing to be concerned about.

Previous work has shown that virtual characters looking towards a participant contributes to Psi. In (Bergström et al. 2017) participants were located close to a virtual string quartet. Psi was greater when the players occasionally looked toward the participant. In (Steed et al. 2018) participants were located on a beach with virtual characters representing refugees waiting for a boat to pick them up. It was found that Psi was greater in a condition when the characters would return glances of the participant towards them compared to a control condition. In (Llobera et al. 2021) participants were amongst a virtual crowd walking towards a theatre. Participants were more likely to choose a condition where the surrounding characters occasionally looked towards themselves rather than one where there was no such feedback. Kyriakou et al. (2017) found that the Psi was increased with respect to a surrounding crowd when a number of realistic crowd behaviours were introduced, including gaze. Gaze behaviour of virtual crowds is important not just when crowd members look towards the participant. Jorjafki et al. (2018) found that even if a small proportion of members of a virtual crowd exhibit gaze behaviour such as looking upwards, then participants will tend to follow their gaze. Ruhland et al. (2015) provide a comprehensive review of eye gaze in virtual characters.

Overall, evidence suggests that gaze of virtual characters towards the participant, whether imagined (Study 1) or programmed (Study 2) is likely to increase the illusion of realness of the events. However, this does not imply that there will be corresponding positive sentiment. In both Study 1 and Study 2 being “stared at” was associated with negative sentiment (even though in Study 1 the virtual characters were not even programmed to look at the participants). Factors that enhance Psi do not necessarily lead to positive sentiment, and there are applications where negative sentiment is the appropriate response, for example in the various studies of bystander response to violent incidents—a recent one reported in (Rovira et al. 2021).

Generating strong Psi is difficult, far more than PI, which depends on sensorimotor contingencies associated with the display device and body tracking. In the case of Psi for a virtual event that is a simulation of real events (such as a rock music performance) meeting expectations is essential. Some of these are obvious, such as the technical capability to match up movements of virtual characters (such as clapping) with the corresponding sounds, making the movements of the drummer or guitarist match with the music, or stopping limbs intersecting bodies. Others depend on deliberate choices of the designers, such as making the audience all appear to be female, or choosing the number and spatial layout of the crowd. Other failures of expectation are more domain specific—such as the clothing and appearance of the virtual crowd not matching the 1980s, or there being no smoking amongst the audience (not fully banned in England in indoor spaces until 2007) and no drinking. To produce a scenario that is Psi-effective it is essential to incorporate as much domain knowledge as possible. This should ideally involve interviews with potential participants, to try to find out what is important to them. Often they will not consciously know this until presented with an actual example and then realize that a particular feature is wrong, or another one is missing. As mentioned earlier, in our bystander studies on soccer violence, it never occurred to us that the decoration of the bar in which the violence took place would be an important factor for Plausibility. This leads to the conclusion that co-design, where potential participants in a VR scenario should be involved from the outset in its design and evaluation, should be employed in the creation of novel scenarios. For example, (García et al. 2021) involved schizophrenia patients in the design and testing of a system for the embodiment of auditory hallucinations in VR. Dietrich et al. (2021) describe two case studies on VR applications for alcohol abuse prevention, and compare different co-design methodologies. Brassel et al. (2021) provide a review of design principles for VR applications in the field of brain injury rehabilitation, and conclude that co-design is an important component.

How much realism is necessary in the rendered scenario for participants to have the illusions of being at a concert (PI) and that the events are really happening (Psi)? There are, of course, many possible meanings of “realism”. This could refer to illumination realism (the lighting), realism of the appearance of the human avatars, realism of animations, and realism of behaviour of the virtual human characters towards the participant. It is noteworthy that in Table 13 there are very few references to the lack of realism of the scenario. On the contrary, Cluster 2 [5] shows one positive comment in this regard. There are some negative comments regarding realism of movement (Cluster 3 [4] and [7]). There are no references in the low sentiment cluster of the lack of illumination realism. There are some references to the lack of realism of animations, and responses of the virtual audience members towards the participant. However, the latter is limited more to the behaviour being socially inappropriate (e.g. “staring”) rather than not being real with respect to their execution. In the early 1990s VR was only capable of rendering scenarios orders of magnitude less realistic than today—the resolution was low, scenarios could have a limited number of polygons, rendering algorithms that included realistic lighting were impossible. Nevertheless, VR was successfully used for the treatment of anxiety disorders—for example, fear of heights (Hodges et al. 1995; Rothbaum et al. 1995a, 1995b), fear of flying (Hodges et al. 1996; Rothbaum et al. 1996), and post-traumatic stress disorder amongst Vietnam veterans (Rothbaum et al. 1999). For such applications to be successful the scenarios had to have had *sufficient realism* to spark anxiety amongst patients during the course of the VR treatment. This implies that the levels of PI and Psi that were generated in these early scenarios were strong enough to obtain these results. Moreover, good illustrations that powerful effects on participants can be achieved in interactions with highly cartoonish virtual human characters are fear of public speaking studies. For example, Pertaub et al. (2002) required participants to give a talk in front of a virtual audience, and they were generally unable to speak coherently when the audience displayed negative behaviours towards them (e.g. showing boredom, yawing, never looking at them, walking out in the middle of the talk, and so on). However, not only were the characters highly cartoonish, but they moved with jerky movements. (A video of this scenario can be seen on https://www.youtube.com/watch?v=rqvdb4gttyU). When the same setup was used to display a highly positive audience participants were well able to give their talk. More recently, in the study mentioned above that placed participants on a shoreline with refugees waiting for a boat (Steed et al. 2018), the characters were deliberately designed to be cartoonish. Nevertheless, the levels of PI and Psi were high, provided that the characters interacted in a minimal way with the participants by returning glances.

McDonnell et al. (2012) carried out a study where 11 different rendering styles were used for virtual human characters ranging from cartoon to more realistic. In psychophysical studies of the effect of the rendering style on participants’ evaluations of lie detection, no critical differences were found between the rendering styles except for characters that were rated in the middle of the “abstract to realistic” scale, since these types of characters were unfamiliar and “difficult for the brain to categorize due to their uncommon experience” (p 91:10). Zibrek et al. (2018) carried out a very large scale study to investigate the interaction between virtual human character rendering styles (“Realistic, Toon CG, Toon Shaded, Creepy and Zombie”, Fig. 2) on affinity towards the characters. The results suggested that the rendering style in itself did not have an effect, only its combination with the type of personality depicted for the character. For example, greater affinity with a realistically rendered character was only in combination with the character depicting neurotic behaviour, but in this case the character was experienced as eerie. Following on from this, Zibrek et al. (2019) in another large-scale experiment studied how virtual human character realism impacted the illusions of PI and copresence (the sense of being with the virtual character), emotional response towards the character, and being in close proximity to the character. Each character expressed being friendly, unfriendly or sad. Again the results were not straightforward. Although greater realism resulted in greater PI, the differences between the conditions had low effect size even if significant—since the level of PI was very high for all of the rendering styles. Copresence was not influenced by rendering style. The lower realism style resulted in greater concern for the character in the sad condition, but in the friendly condition the higher realism style led to greater concern for the character. Rendering style did not influence the effects of proximity, only the emotional expression of the character. Finally in this series of studies Zibrek and McDonnell (2019) embodied participants in a photorealistic virtual body and they then interacted with another character rendered either simplistically or in a photorealistic style. The results showed higher PI and copresence for the realistic character, no effect with respect to being close to the character, but concern for the character dependent on the order of presentation of the conditions in this within groups experiment. However, the result is open to interpretation, since possibly the inconsistency between the photorealistic body used for embodiment and the lower realism of the other character in the simplified style could have played a part in these results.

What this series of studies illustrates above all is that there is no simple equation: that higher realism results in greater PI or Psi, or other factors considered by those authors. Prior to our experiment we were concerned that participants would simply reject the concert scenario due to its evident lack of fidelity to a real concert. However, this did not happen. Participants demonstrated high levels of Psi, as evidenced by their negative sentiment towards some of the actions of the audience around them (in particular “staring”). Moreover, Fig. 1 shows the level of realism of the characters which could be argued to be quite high. However, since we did not do a comparative study of different levels of realism, we cannot know if the results would have changed for either lower or greater degrees of visual or behavioural realism. The contribution of this paper is not with respect to the methods used to create the scenario, or particular rendering and animation techniques, but rather with respect to the method of evaluation. Given *this particular rendition of the concert*, how would participants respond? We now return to the issue of the evaluation method.

While there has been significant use of sentiment analysis in a wide variety of domains—see (Alamoodi et al. 2021; Birjali et al. 2021)—it has seen little use in the design and evaluation of VR applications. Fagernäs et al. (2021) point out that although there have been experimental studies with participants in the field of VR for the promotion of relaxation techniques, sentiment analysis can be used to find out what users actually think of these techniques, and point the way to improvements in applications. As mentioned earlier, when we carry out experimental studies essentially we are testing the models of the researchers involved, not necessarily what is important to participants. For decades, VR researchers (including ourselves) have concentrated heavily on presence (as “being there”) but it is possible that this concept is imposed on participants in experimental studies through the questions that they are required to answer (Slater, 2004). However, something more basic, and perhaps obvious than “being there” is simply to follow the preferences of participants. We have started to employ this methodology in recent work—in (Murcia-López et al. 2020) participants experienced a talk from a virtual character and were able to select different options to change the characteristics of the character and aspects of the setting in real-time throughout the session to match their preferences. In (Llobera et al. 2021) the same technique was used, except that the possible changes available to participants were proposed by a reinforcement learning agent. However, in work that has used this methodology, for example recently (Fribourg et al. 2020), the possible changes that can be made to the environment are fixed and chosen in advance by the researchers. We suggest that the use of sentiment analysis as a methodology to understand how participants respond to VR applications would be a useful way forward in the co-design process of building applications that are more likely to be preferred. This does not necessarily mean designing applications that result in positive sentiment, since negative sentiment, for example in psychological therapy applications, might be part of the goal. Rather the idea is to uncover features of the virtual environment that unexpectedly result in negative or inappropriate sentiment, and in the next phase of application development, overcome those problems.

The methodology adopted in this paper has illustrated the power of this type of qualitative to quantitative analysis to give deep insight into the responses of people to the virtual reality scenario. If we would have just followed traditional approaches we would have had answers to some fixed questions and Likert scale scores, which also have important problems with respect to interpretation and analysis (Slater and Garau 2007). We would not have known the features that enhanced or detracted from the illusion of being at a concert, only a set of scores that scratch the surface of the responses of people. We have discovered that the overwhelming reaction to the concert is not so much the performance of the band but the quality of the audience, and the relationship between the audience and the participant.

The resulting analysis has pointed out several features that we must pay attention to in subsequent versions of our applications. *The participant is alone*—which can be overcome by supporting several friends to simultaneously attend the concert. *The crowd consists of individuals rather than groups of friends*—a future version of the concert needs to depict the crowd as consisting of groups who seem to be together. *The characters around stare at the participant for too long*—using data about glance times could help to overcome this problem. *Audience members do not interact with the participant except for gaze*—research into how members of a concert audience actually interact is essential. *The lack of diversity of the crowd is a major issue*—this was a deliberate choice for experimental reasons but is being addressed in current work. *The clothing and behaviour of the virtual audience does not match the time at which the real concert took place* (1983)—research on this issue would be required to properly match these expectations. *The band does not interact with the audience nor the audience with each other*—this is mainly due to the fact that the entire video of the “Sultans of Swing” recording was not transformed into animated 3D, which would have displayed the actual interactions between members of the band. *There are multiple technical problems such as the lack of sync between movements of the crowd members and the corresponding sounds, and also the musicians and the song*—this requires improvements in the implementation. *The audio aspect was well considered but the sounds of the crowd around need to originate from close distance rather than blended in with the sound that appears to be coming from the stage*—the sound recording needs to be separated into different streams, the band and the audience sound, but also sounds from the immediate surrounding audience members need to be incorporated into the scenario. These issues all have mainly technical solutions that can be addressed in our future work.

The lack of synchrony between movement and sound should, a priori, have been a major distracting factor. However, in Study 1 (Table 6) there were at most 6 entries about synchrony failures (Cluster 2 [2], Cluster 2 “realistic crowd”, Cluster 2 [3], Cluster 3 [4], Cluster 3 “ok clapping”, Cluster 4 “stationary hand”), but one positive comment (Cluster 4 “nice move”). In Table 13 there are 3 references to the lack of synchrony (Cluster 1 [8], Cluster 2 [13], Cluster 3 [11]. The interesting question that follows is why there were not more complaints about the lack of synchrony? In the video extract https://youtu.be/bOSWaKT88j4 we show the occasions when the audience was cheering or clapping. Overall, regarding the cheering, the movements of the virtual audience and the sound are mostly correlated. However, in the case of the clapping, although the movements correlate overall, the clapping itself is inaccurate with the hands not quite touching together at the moment of the clap. Possibly this did not become a major issue because there is so much going on in the scenario and the failure of the clapping occupied only a small portion of the overall scenario. However, more striking is the lack of correspondence between the detailed movements of the band playing their instruments and the corresponding sounds. For example, the drummer is only accidentally in time with the beat, and the lead guitarist did not slide his fingers up the neck of the guitar in order to produce the higher notes. All of these things become clear on deliberate observation and reflection during the scenario, which, however, may be missed in the excitement of the overall performance, or the discomfort caused by surrounding audience members. Moreover, Petrini et al. (2009) in a study of visual-auditory synchrony detection in the context of drumming, found that musical expertise played a role—the greater the expertise the more likely that asynchrony would be detected. In future studies obtaining background information on the extent to which participants had musical knowledge, and their frequency of concert going would be important.

In general many of the issues above, such as lack of diversity, will be important for VR applications that include multiple virtual human characters that are simultaneously influenced by external factors—in our case the band and the music, but other examples might include depictions of urban settings where the crowd members are influenced by one another, and by traffic. An example of people at a train station, where some of them start running, that then influences the participants, is given in (Ríos and Pelechano 2020). The appearance of a crowd attending a scenario in VR should be based on prior studies about how the composition of such a crowd would be in reality. This adds to the point about co-design, and in particular the need to show early versions of a system to potential participants in order to obtain these comments. This is an important point illustrated by the current paper—our version of the concert was shown to others via these experimental studies and lack of diversity was one of the issues that came up that need to be addressed in future versions.

The Likert scale was introduced in the 1930s, nearly a century ago. It is time that the incredible developments in machine learning and analysis of natural language are brought into the domain of experimental studies. The “meta message” of this paper is to bring VR experimental studies into the 21st Century.

## Supplementary Information

Below is the link to the electronic supplementary material.

Supplementary file1 (DOCX 16 kb)

## Footnotes

1 www.qualtrics.com.

2 https://github.com/trinker/sentimentr#comparing-sentimentr-syuzhet-meanr-and-stanford.

3 https://CRAN.R-project.org/package=vader.

4 https://github.com/mjockers/syuzhet.

5 https://CRAN.R-project.org/package=SentimentAnalysis.

6 https://cran.r-project.org/web/packages/factoextra/index.html.

7 https://CRAN.R-project.org/package=udpipe.

8 http://www.bnosac.be/index.php/blog/77-an-overview-of-keyword-extraction-techniques.

9 https://www.rdocumentation.org/packages/lexRankr/versions/0.5.2/topics/lexRank.
