# Supplementary material for: The sentiment of a virtual rock concert
Source: Virtual Real. 2022 Aug 23;27(2):651–75. doi: 10.1007/s10055-022-00685-9 (PMC9396608; doi:10.1007/s10055-022-00685-9)
Supplement: Supplementary file 1 — Supplementary file1 (DOCX 16 kb) [file 10055_2022_685_MOESM1_ESM.docx]

The Sentiment of a Virtual Rock Concert

Mel Slater^1,2^, Carlos Cabriera^1^, Gizem Senel^1,2^, Domna Banakou^1,2^,
Alejandro Beacco^1^, Ramon Oliva^1^, Jaime Gallego^1^

^1^ Event Lab, Faculty of Psychology, University of Barcelona, Barcelona, Spain

2 Institute of Neurosciences of the University of Barcelona, Barcelona, Spain

# Supplementary Information

## Supplementary Table S1 - Characteristics of the Sample

|  | **Study 1** | **Study 2** | **Overall** |
| --- | --- | --- | --- |
| *n* | 25 | 26 | 51 |
| *Gender* | M: 8  F:17 | M: 19  F: 7 | M: 27  F: 24 |
| *Age*  18-24 = 1  25-34 = 2  35-44 = 3  45-54 = 4  55-64 = 5  65-74 = 6  75 and over = 7 | 1: 8  2: 8  3: 4  4: 4  5: 0  6: 1  7: 0  Median: 2  IQR: 1 - 3 | 1: 9  2: 6  3: 4  4: 6  5: 0  6: 1  7: 0  Median: 2  IQR: 1 – 3.75 | 1: 17  2: 14  3: 8  4: 10  5: 0  6: 1  7: 0  Median: 2  IQR: 1 – 3 |
| *Group*  Classes (US, Australia):  Classes (MIT):  Social Media: | 25 | 12  5  9 | 12  30  9 |
| *HMD*  Quest 1:  Quest 2:  Pico: | 25 | 5  16  5 | 30  16  5 |
| *What is your technical expertise in virtual reality?*  Each of the 3 questions below are on a 1-7 scale where  1 = Not at all  4 = Some experience  7 = Expert |  |  |  |
| *vrprogram:*  I program virtual reality experiences. |  | 1: 1  2: 6  3: 1  4: 10  5: 4  6: 2  7: 2  Median = 4  IQR: 2.25 - 5 |  |
| *vrdesign:*  I design virtual reality experience. |  | 1: 1  2: 5  3: 1  4: 7  5: 5  6: 5  7: 2  Median: 4  IQR: 3.25 – 5.75 |  |
| *vreval:*  I evaluate virtual reality experiences. |  | 1: 2  2: 3  3: 1  4: 10  5: 4  6: 5  7: 1  Median: 4  IQR: 4 - 5 |  |
| *Condition*   \| *Gaze/Crowd* \| *F* \| *M* \| \| --- \| --- \| --- \| \| *NoLookAt* \| *0* \| *2* \| \| *LookAt* \| *1* \|  \| | 0:  1:  2: 25 | 0: 15  1: 11  2: | 0: 15  1: 11  2: 25 |
